# Levels of vaccination coverage among HIV-exposed children in China: a retrospective study

**DOI:** 10.1186/s40249-021-00797-5

**Published:** 2021-03-01

**Authors:** Rui Shen, Ai-Ling Wang, Xiao-Ping Pan, Ya-Ping Qiao, Qian Wang, Xiao-Yan Wang, Shui-Ling Qu, Tong Zhang

**Affiliations:** 1 National Center for Women and Children’s Health, Chinese Center for Disease Control and Prevention, No. 12 Dahuisi Road, Haidian District, Beijing, 100081 China; 2 Chinese Center for Disease Control and Prevention, 155 Changbai Road, Changping District, Beijing, 102206 China; 3 Capital Institute of Pediatrics, No. 2 Yabao Road, Chaoyang District, Beijing, 100020 China

**Keywords:** HIV, Children, Coverage, Vaccination, China

## Abstract

**Background:**

Vaccination is crucial for human immunodeficiency virus (HIV)-exposed children because of their increased risk of morbidity and mortality from various vaccine-preventable diseases. However, studies have shown that they are at high risk of incomplete vaccination. Although China has developed prevention of mother-to-child transmission (PMTCT) of HIV programs substantially over the past decades, few studies have investigated the immunization levels of Chinese HIV-exposed children. Therefore, we aimed to evaluate vaccination coverage and its associated factors among HIV-exposed children in China during 2016‒2018.

**Methods:**

We conducted a retrospective cohort review of all cases of Chinese HIV-exposed children born between July 1, 2016 and June 30, 2018 recorded in the Chinese information system on PMTCT. The vaccination coverage indicators refer to the percentage of children who received recommended basic vaccines, including Bacillus Calmette-Guérin (BCG), hepatitis B (HepB), polio, measles-containing vaccine (MCV), and diphtheria-tetanus-pertussis-containing (DTP) vaccine. Univariate and multivariate logistic regression analyses expressed as crude odds ratios (c*OR*s) and adjusted odds ratios (a*OR*s), each with 95% confidence intervals (95% *CI*), were performed to compare the proportional differences of factors associated with vaccine coverage.

**Results:**

Among the enrolled 10 033 children, the vaccination rate was 54.1% for BCG, 84.5% for complete HepB vaccination, 54.5% for complete polio vaccination, 51.3% for MCV, and 59.5% for complete DTP vaccination. Children with perinatally acquired HIV (PHIV) were 2.46‒3.82 times less likely to be vaccinated than HIV-exposed uninfected children. Multivariate logistic regression indicated that children of Han ethnicity (a*OR* = 1.33‒2.04), children with early infant diagnosis (EID) of HIV (a*OR* = 1.86‒3.17), and children whose mothers had better education (college or above, a*OR* = 1.63‒2.51) had higher odds of being vaccinated. Most of the deceased children (a*OR* = 4.28‒21.55) missed vaccination, and PHIV (a*OR* = 2.46‒3.82) significantly affected immunization.

**Conclusions:**

Chinese HIV-exposed children had low vaccination coverage, which is a serious health challenge that needs to be addressed thoroughly. Interventions should be developed with a focus on minority HIV-exposed children whose mothers do not have formal education. Particularly, more attention should be paid to EID to increase access to immunization.

**Graphical abstract:**

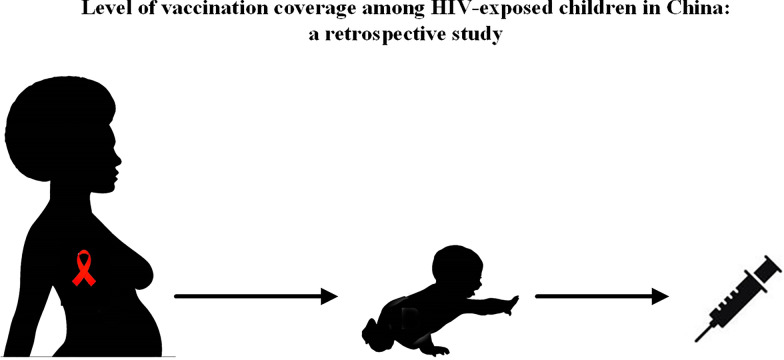

## Background

Immunization has been identified as one of the most cost-effective interventions in global health. The World Health Organization (WHO) monitors vaccine coverage as a key indicator of children's health services [1]. However, studies have shown that children born to human immunodeficiency virus (HIV)-infected women have 30–70% chance of being incompletely immunized [2–4]. Compared to infants of uninfected mothers, these children are reported to have a markedly higher early life burden of infectious diseases for their immunological disorders [5, 6]. Low vaccination coverage may increase the morbidity and mortality risk from vaccine-preventable diseases among such children [7–9].

With the effectiveness of prevention of mother-to-child transmission (PMTCT) interventions well established, China has reduced the mother-to-child transmission rate of HIV to 4.9% in 2017 [10]. Most children of Chinese HIV-infected mothers are not infected with HIV, but the mortality rate among those aged 0‒18 months is as high as 4.9‒7.8% [11–13], which is significantly higher than the Chinese national infant mortality rate of 0.61% [14]. Infectious diseases remain a major cause of death among HIV-exposed children [12, 15, 16].

Previous studies indicated that one reason for increased mortality among children born to HIV-positive mothers might be that those children are less likely to receive routine childhood vaccinations [4, 17]. However, few studies have reported immunization levels in HIV-exposed Chinese children. Given the importance of vaccination for HIV-exposed children and the data from other countries, suggesting that they are at a higher risk of under-vaccination, we evaluated the vaccination rates and factors affecting the vaccination status of HIV-exposed children in China.

## Methods

### Study design and setting

This was a retrospective cohort study using data from the Chinese information system for PMTCT. This longitudinal, population-based health and vital event registration system monitors HIV-exposed children and their mothers. It is implemented through mandatory case reporting by health facilities in all 31 provincial-level administrative divisions of China. For each child, data collected include maternal demographic characteristics (e.g., maternal age, occupation, education, ethnicity, marital status, and parity), child’s characteristics (e.g., sex, birth weight, and HIV status), and clinically standardized follow-up information (HIV laboratory assessment, vaccination history, HIV-related diseases and symptoms, and survival status). These data are prospectively collected both at birth and at 1, 3, 6, 9, 12, 15, and 18 months of age [13, 18, 19].

### Study population

The target population in the study was perinatally HIV-exposed children, with the following criteria: (1) born to HIV-infected women, (2) live birth, and (3) born between July 1, 2016 and June 30, 2018. Children who were lost to follow-up were excluded. All the appropriate cases in the PMTCT information system were included in this study.

### Data source and variables

Variables and data related to vaccination status were extracted from PMTCT information system—belongs to China CDC, which is not open for the public—using a standardized data abstraction form. The study variables included maternal age, ethnicity, marital status, parity, education, and occupation; and the child’s gender, birth weight, HIV status, HIV-related diseases (pathological jaundice, upper respiratory tract infection, pathological diarrhea, pneumonia, anemia, rickets, or severe malnutrition), HIV-related symptoms (intermittent or persistent fever, persistent cough, rash, systemic lymphadenopathy, *Candida albicans* infection, or hepatosplenomegaly), and survival status (alive or dead). HIV infection was diagnosed according to reactive HIV tests on two different occasions: positive virological tests under 18 months of age or positive antibody tests up to 18 months. Early infant diagnosis (EID) is used to diagnose babies born to HIV-infected mothers as recommended by the WHO. In our study, HIV-exposed children undergo EID at 42 days and 3 months of age to confirm HIV status by HIV virological tests. According to the WHO [20] and the Chinese National Immunization Programme schedule [21], vaccination coverage indicators refer to the percentage of children who received recommended basic vaccines, including one dose of Bacillus Calmette-Guérin (BCG) vaccine, three doses of hepatitis B (HepB) vaccine, three doses of polio vaccine (live oral poliovirus vaccine or inactivated poliovirus vaccine), one dose of measles-containing vaccine (MCV), and three doses of diphtheria-tetanus-pertussis-containing (DTP) vaccine. Children were classified as up to date (UTD) if they had received the recommended dose of vaccines before 12 months of age.

### Immunization schedule

According to the Chinese immunization guidelines [21] (Table 1), children born to HIV-infected mothers should not be vaccinated with oral poliomyelitis vaccine (OPV) and BCG; these vaccinations should be postponed until the children are confirmed as not infected with HIV. Children with HIV-related or immunosuppressive symptoms should not be vaccinated with MCV. HIV-exposed uninfected (HEU) infants are recommended to have immunization as is routinely administered to other children, including one dose of BCG after birth, three doses of HepB vaccine (at birth, 1 month, and 6 months of age), three doses of polio vaccine (at 2, 3, and 4 months of age), three doses of DTP vaccine (at 3, 4, and 5 months of age), and one dose of MCV (at 8 months).

**Table 1:** Chinese immunization recommendations for HIV-exposed infants

Vaccines	HIV-infected infants		Unknown HIV status		HIV-uninfected infants
	Have symptoms or immunosuppression	Asymptomatic and non-immunosuppressive	Have symptoms or immunosuppression	Asymptomatic	
BCG	×	×	Postponed	Postponed	√
HepB	√	√	√	√	√
IPV	√	√	√	√	√
OPV	×	×	×	×	√
MCV	×	√	×	√	√
DTP	√	√	√	√	√

*BCG* Bacillus-Calmette-Guérin, *DTP* diphtheria, tetanus, and pertussis, *HepB* hepatitis B, *HIV* human immunodeficiency virus, *IPV* inactivated poliovirus vaccine, *MCV* measles-containing vaccine, *OPV* oral poliovirus vaccine

√:Recommended

× : Not recommended

### Statistical analysis

Statistical analysis was performed using the statistical software SPSS 25.0 (International Business Machines Corporation, Armonk, USA). Immunization status was presented as numbers and percentages. Proportions were compared by Chi-square tests. Univariate and multivariate logistic regression analyses were performed to compare the proportional differences in factors associated with vaccination coverage. Univariate logistic regression was used for categorical variables to explore potential factors affecting immunization. Multivariate logistic regression using the forward likelihood ratio method was utilized to analyze factors associated with non-uptake of vaccines among the studied population. Crude odds ratio (c*OR*) and adjusted odds ratio (a*OR*) were calculated with logistic regression. Significance tests were two-tailed, and *P* values < 0.05 were considered statistically significant.

## Results

### Overall characteristics of the population

We included 10 033 live-born HIV-exposed children born from July 1, 2016 to June 30, 2018, excluding 119 children lost to follow-up. The characteristics of the enrolled children and their mothers are reported in Table 2. There were 306 children with perinatally acquired HIV (PHIV), 341 with unknown HIV status, and 9386 HEU children among the 10 033 enrolled children. We found that 49.5% of the children were of Han ethnicity, 89.6% had EID to determine HIV status, 11.6% had at least one HIV-related symptom, and 17.0% had at least one HIV-related disease during the 18 months of follow-up. In all, 3.0% of the children died during the 18 months of follow-up. Most of the HIV-infected mothers lived with a spouse (92.7%), were aged 20–34 years (77.9%), graduated from middle school (48.8%), farmed at home (56.1%), and had given birth to 2–3 babies (55.9%).

**Table 2:** Maternal and child characteristics in the study population

Variables	Total *N* = 10 033	HIV status			*P-*value^a^
		HEU	PHIV	Unknown	
	*N* (%)	*n* (%)	*n* (%)	*n* (%)	
Sex					0.028
Male	5168 (51.5)	4864 (51.8)	136 (44.4)	168 (49.3)	
Female	4865 (48.5)	4522 (48.2)	170 (55.6)	173 (50.7)	
Birth weight (g)					0.030
< 2500	1261 (12.6)	1161 (12.4)	38 (12.4)	62 (18.2)	
2500‒4000	8607 (85.8)	8073 (86.0)	258 (84.3)	276 (80.9)	
> 4000	165 (1.6)	152 (1.6)	10 (3.3)	3 (0.9)	
EID					< 0.001
Yes	8994 (89.6)	8680 (92.5)	295 (96.4)	19 (5.6)	
No	1039 (10.4)	706 (7.5)	11 (3.6)	322 (94.4)	
HIV-related diseases					< 0.001
No	8329 (83.0)	7995 (85.2)	174 (56.9)	160 (46.9)	
Yes	1704 (17.0)	1391 (14.8)	132 (43.1)	181 (53.1)	
HIV-related symptoms					< 0.001
No	8870 (88.4)	8500 (90.6)	199 (65.0)	171 (50.1)	
Yes	1163 (11.6)	886 (9.4)	107 (35.0)	170 (49.9)	
Survival status					< 0.001
Live	9730 (97.0)	9285 (98.9)	235 (76.8)	210 (61.6)	
Dead	303 (3.0)	101 (1.1)	71 (23.2)	131 (38.4)	
Maternal age (years)					0.022
< 20	345 (3.5)	314 (3.3)	10 (3.3)	21 (6.2)	
20‒34	7819 (77.9)	7329 (78.1)	227 (74.2)	263 (77.1)	
≥ 35	1869 (18.6)	1743 (18.6)	69 (22.5)	57 (16.7)	
Ethnicity					0.006
Han	4963 (49.5)	4676 (49.8)	125 (40.8)	162 (47.5)	
Minority	5070 (50.5)	4710 (50.2)	181 (59.2)	179 (52.5)	
Marital status					0.017
Single	730 (7.3)	665 (7.1)	29 (9.5)	36 (10.6)	
Married/cohabitated	9303 (92.7)	8721 (92.9)	277 (90.5)	305 (89.4)	
Education					< 0.001
Primary or lower	4255 (42.4)	3936 (41.9)	160 (52.3)	159 (46.6)	
Middle school	4897 (48.8)	4617 (49.2)	128 (41.8)	152 (44.6)	
College or above	700 (7.0)	674 (7.2%)	12 (3.9)	14 (4.1)	
Unknown	181 (1.8)	159 (1.7)	6 (2.0)	16 (4.7)	
Occupation					0.083
Farmer	5628 (56.1)	5295 (56.4)	165 (53.9)	168 (49.3)	
Housewife/unemployed	2678 (26.7)	2488 (26.5)	89 (29.1)	101 (29.6)	
Others	1727 (17.2)	1603 (17.1)	52 (17.0)	72 (21.1)	
Parity					0.086
1	1981 (19.8)	1829 (19.5)	67 (21.9)	85 (24.9)	
2–3	5610 (55.9)	5259 (56.0)	165 (53.9)	186 (54.6)	
> 3	2442 (24.3)	2298 (24.5)	74 (24.2)	70 (20.5)	

*EID* early infant diagnosis, *HEU* HIV-exposed uninfected, *HIV* human immunodeficiency virus, *N* number of children, *n*: number of children with the characteristic, *PHIV* perinatally acquired HIV, children perinatally infected with HIV from mothers

^a^Chi-square test

### Vaccination status

#### Vaccination coverage

For the 10 033 children included in the analysis, the BCG vaccination rate was 54.1%, excluding 150 cases with unknown BCG vaccination status. The vaccination rates for three injections of HepB were respectively 97.1, 89.9, and 84.5%, excluding 131 cases of missing HepB vaccination data. The first dose of the polio vaccine was received by 63.5% of the children, the second by 58.7%, and the third by 54.5%, excluding 206 cases of insufficient reporting. MCV rate was 51.3%, excluding 211 cases of unknown vaccination status; DTP vaccination rate was 66.3% for the first dose, and the complete vaccination rate for three injections was 59.5%, excluding 212 cases of insufficient data. Compared with HEU children, children with PHIV were less likely to be vaccinated with BCG (28.9 vs 55.7%), HepB1 (90.5 vs 98.0%), Polio1 (33.0 vs 65.7%), MCV (19.7 vs 53.4%), and DTP1 (37.6 vs 68.5%). Children with PHIV were also less likely to have received the second and third doses of HepB, polio, and DTP vaccines than HEU children (Table 3).

**Table 3:** Vaccination coverage of five basic vaccines in the study population

Vaccines	Total *N*	Vaccinated children *n* (%)	HIV status			*P-*value^a^
			HEU *n* (%)	PHIV *n* (%)	Unknown *n* (%)	
BCG	9883	5346 (54.1)	5192 (55.7)	88 (28.9)	66 (25.3)	< 0.001
HepB1	9902	9611 (97.1)	9141 (98.0)	276 (90.5)	194 (72.1)	< 0.001
HepB2	9902	8901 (89.9)	8583 (92.0)	223 (73.1)	95 (35.3)	< 0.001
HepB3	9902	8370 (84.5)	8149 (87.4)	163 (53.4)	58 (21.6)	< 0.001
Polio1	9827	6236 (63.5)	6092 (65.7)	100 (33.0)	44 (17.5)	< 0.001
Polio2	9827	5772 (58.7)	5657 (61.0)	78 (25.7)	37 (14.7)	< 0.001
Polio3	9827	5354 (54.5)	5253 (56.6)	67 (22.1)	34 (13.5)	< 0.001
MCV	9822	5044 (51.3)	4951 (53.4)	60 (19.7)	33 (13.1)	< 0.001
DTP1	9821	6514 (66.3)	6349 (68.5)	114 (37.6)	51 (20.2)	< 0.001
DTP2	9821	6210 (63.2)	6069 (65.5)	100 (33.0)	41 (16.3)	< 0.001
DTP3	9821	5842 (59.5)	5714 (61.7)	89 (29.4)	39 (15.5)	< 0.001

*BCG* Bacillus-Calmette-Guérin, *DTP* diphtheria-tetanus-pertussis-containing, *HEU* HIV-exposed uninfected, *HIV* human immunodeficiency virus, *HepB* hepatitis B, *MCV* measles-containing vaccine, *N* number of children, *n* number of children with the characteristic, *PHIV* perinatally acquired HIV, children perinatally infected with HIV from mothers

^a^Chi-square test

#### Vaccination up to date status

HEU children were significantly (*P* < 0.05) more likely to be UTD for each vaccine by 12 months of age, and the probability of being UTD for HEU children was 3.00‒4.65 times more than that of children with PHIV (Table 4). Differences in UTD status between PHIV and HEU groups were large: BCG (27.0 vs 52.6%), HepB3 (50.8 vs 82.8%), Polio3 (19.1 vs 51.3%), MCV (17.1 vs 48.6%), and DTP3 (25.4 vs 55.5%). Only 9.3% of children with PHIV had received all five routine vaccines at 12 months of age, compared to 34.4% of HEU children (Table 5).

**Table 4:** Vaccination up to date status in the study population

Vaccine	Total *N*	UTD *n* (%)	PHIV		HEU		Unknown	
			*n* (%)	*OR* (95% *CI*)	*n* (%)	*OR* (95% *CI*)	*n* (%)	*OR* (95% *CI*)
				*P*-value^a^		*P*-value^a^		*P*-value^a^
BCG	9883	5041 (51.0)	82 (27.0)	1.00 –	4 900 (52.6)	3.00 (2.32‒3.88) < 0.001	59 (22.6)	0.79 (0.54‒1.16) 0.232
HepB3	9902	7928 (80.1)	155 (50.8)	1.00 –	7 720 (82.8)	4.65 (3.69‒5.85) < 0.001	53 (19.7)	0.24 (0.16‒0.35) < 0.001
Polio3	9827	4848 (49.3)	58 (19.1)	1.00 –	4 758 (51.3)	4.45 (3.33‒5.94) < 0.001	32 (12.7)	0.62 (0.39‒0.99) 0.044
MCV	9822	4582 (46.6)	52 (17.1)	1.00 –	4 504 (48.6)	4.58 (3.39‒6.20) < 0.001	26 (10.3)	0.56 (0.34‒0.92) 0.023
DTP3	9821	5257 (53.5)	77 (25.4)	1.00 –	5 144 (55.5)	3.66 (2.82‒4.76) < 0.001	36 (14.3)	0.49 (0.32‒0.76) 0.001

*BCG* Bacillus-Calmette-Guérin, *CI* confidence interval, *DTP* diphtheria-tetanus-pertussis-containing, *HEU* HIV-exposed uninfected, *HIV* human immunodeficiency virus, *HepB* hepatitis B, *MCV* measles-containing vaccine, *N* number of children, *n* number of children with the characteristic, *OR* odds ratio, *PHIV* perinatally acquired HIV, children perinatally infected with HIV from mothers, *UTD* up to date, children were classified as up to date if they had received the recommended dose of vaccines before 12 months of age. – means not applicable

^a^Univariate logistic regression

**Table 5:** Number of vaccines up to date in the study population

Number of vaccines UTD	Total *N* = 10,033 *n* (%)	PHIV		HEU		Unknown	
		*n* (%)	*OR* (95% *CI*) *P*-value^a^	*n* (%)	*OR* (95% *CI*) *P*-value^a^	*n* (%)	*OR* (95% *CI*) *P*-value^a^
None UTD^b^	1462 (14.9)	124 (40.9)	1.00	1164 (12.6)	1.00 –	174 (70.2)	1.00 –
1–4 UTD	5107 (52.1)	151 (49.8)	1.00	4904 (53.0)	3.46 (2.71‒4.43) < 0.001	52 (21.0)	0.25 (0.17‒0.36) < 0.001
All UTD^c^	3230 (33.0)	28 (9.3)	1.00	3180 (34.4)	12.10 (7.99‒18.33) < 0.001	22 (8.9)	0.56 (0.31‒1.02) 0.06
Missing or insufficient data	234	3	–	138	–	93	–

*CI* confidence interval, *HEU* HIV-exposed uninfected, *N* number of children, *n* number of children with the characteristic, *OR* odds ratio, *PHIV* perinatally acquired HIV, children perinatally infected with HIV from mothers, *UTD* up to date, children were classified as up to date if they had received the recommended dose of vaccines before 12 months of age; – means not applicable

^a^Univariate logistic regression;

^b^None UTD: None of the targeted vaccines was UTD at 12 months old;

^c^All vaccines UTD include: BCG, Hep B, MCV, DTP and Polio

#### Vaccination factors

In univariate analysis, there were significant (*P* < 0.05) associations of child characteristics (survival status, HIV status, HIV-related symptoms, and EID) and maternal features (ethnicity, education, and occupation) with vaccination status (Table 6). Marital status and HIV-related diseases varied among those who received vaccines. Vaccine status had no statistical (*P* > 0.05) association with child’s sex and birth weight, and maternal age and parity. HEU children were significantly (*P* < 0.05) more likely to be vaccinated than children with PHIV. In the multivariate analysis, five characteristics (maternal education, maternal ethnicity, child’s HIV status, child survival status, and whether or not EID was done) remained independently associated with vaccination status after controlling covariates (Table 7). Most of the dead children missed vaccination (a*OR* = 4.28‒21.55). HIV status (HEU vs PHIV, a*OR* = 2.46‒3.82) and EID (a*OR* = 1.86‒3.17) played significantly crucial roles in vaccination, as did ethnicity (Han vs minority, a*OR* = 1.33‒2.04). For other variables, children of housewives/unemployed mothers had higher odds of being vaccinated than those of farmers for Polio3 [a*OR* = 1.15, 95% confidence interval (*CI*): 1.03‒1.28], MCV (a*OR* = 1.15, 95% *CI*: 1.03‒1.28), and DTP3 (a*OR* = 1.39, 95% *CI*: 1.24‒1.55).

**Table 6:** Crude odds of vaccination factors among HIV-exposed children

Variable	BCG		Polio3		DTP3		HepB3		MCV	
	c*OR* (95%* CI*)	*P*-value^a^	c*OR* (95% *CI*)	*P*-value^a^	c*OR* (95%* CI*)	*P*-value^a^	c*OR* (95% *CI*)	*P*-value^a^	c*OR* (95% *CI*)	*P*-value^a^
Sex										
Male	1.00		1.00		1.00		1.00		1.00	
Female	1.04 (0.96‒1.12)	0.384	1.06 (0.98‒1.15)	0.152	1.00 (0.93‒1.09)	0.905	1.07 (0.96‒1.19)	0.221	1.05 (0.97‒1.14)	0.189
EID										
No	1.00		1.00		1.00		1.00		1.00	
Yes	2.40 (2.08‒2.76)	< 0.001	3.42 (2.95‒3.97)	< 0.001	3.87 (3.34‒4.48)	< 0.001	5.38 (4.66‒6.20)	< 0.001	3.32 (2.85‒3.87)	< 0.001
HIV-related diseases										
Yes	1.00		1.00		1.00		1.00		1.00	
No	0.97 (0.87‒1.08)	0.570	0.97 (0.87‒1.08)	0.608	1.04 (0.93‒1.15)	0.529	1.78 (1.56‒2.03)	< 0.001	0.91 (0.82‒1.02)	0.094
HIV-related symptoms										
Yes	1.00		1.00		1.00		1.00		1.00	
No	1.19 (1.05‒1.35)	0.008	1.37 (1.21‒1.56)	< 0.001	1.54 (1.36‒1.75)	< 0.001	2.66 (2.31‒3.07)	< 0.001	1.24 (1.09‒1.40)	0.001
Survival status										
Dead	1.00		1.00		1.00		1.00		1.00	
Live	9.07 (6.33‒13.01)	< 0.001	49.46 (23.33‒104.84)	< 0.001	42.45 (22.55‒79.91)	< 0.001	54.22 (37.18‒79.07)	< 0.001	30.03 (15.96‒56.53)	< 0.001
Birth weight (g)										
< 2500	1.00		1.00		1.00		1.00		1.00	
2500–4000	1.01 (0.90‒1.14)	0.814	0.97 (0.86‒1.09)	0.571	0.91 (0.80‒1.03)	0.125	1.13 (0.97‒1.33)	0.125	0.99 (0.88‒1.12)	0.882
> 4000	1.35 (0.97‒1.88)	0.078	0.97 (0.70‒1.35)	0.879	0.80 (0.58‒1.11)	0.188	1.08 (0.69‒1.69)	0.728	1.24 (0.89‒1.72)	0.202
HIV status										
PHIV	1.00		1.00		1.00		1.00		1.00	
HEU	3.09 (2.40‒3.97)	< 0.001	4.60 (3.50‒6.06)	< 0.001	3.87 (3.01‒4.97)	< 0.001	6.02 (4.77‒7.60)	< 0.001	4.67 (3.51‒6.21)	< 0.001
Unknown	0.83 (0.57‒1.21)	0.330	0.55 (0.35‒0.87)	0.010	0.44 (0.29‒0.67)	< 0.001	0.24 (0.17‒0.35)	< 0.001	0.61 (0.39‒0.97)	0.038
Maternal age (years)										
< 20	1.00		1.00		1.00		1.00		1.00	
20‒34	0.86 (0.69‒1.07)	0.178	0.90 (0.72‒1.12)	0.356	0.95 (0.76‒1.18)	0.636	1.08 (0.80‒1.45)	0.620	0.83 (0.67‒1.03)	0.097
≥ 35	0.83 (0.66‒1.05)	0.119	0.92 (0.73‒1.17)	0.508	0.99 (0.78‒1.26)	0.937	1.01 (0.74‒1.38)	0.946	0.81 (0.64‒1.02)	0.074
Ethnicity										
Minority	1.00		1.00		1.00		1.00		1.00	
Han	2.60 (2.40‒2.82)	< 0.001	2.19 (2.02‒2.38)	< 0.001	2.62 (2.41‒2.84)	< 0.001	1.79 (1.60‒2.01)	< 0.001	2.16 (2.00‒2.34)	< 0.001
Marital status										
Married/Cohabitated	1.00		1.00		1.00		1.00		1.00	
Single	1.25 (1.07‒1.46)	0.004	1.32 (1.13‒1.55)	< 0.001	1.48 (1.26‒1.74)	< 0.001	0.99 (0.80‒1.22)	0.941	1.19 (1.02‒1.38)	0.029
Education										
Primary or lower	1.00		1.00		1.00		1.00		1.00	
Middle school	2.36 (2.17‒2.57)	< 0.001	2.43 (2.23‒2.64)	< 0.001	3.05 (2.80‒3.33)	< 0.001	2.02 (1.80‒2.27)	< 0.001	2.22 (2.04‒2.41)	< 0.001
College or above	2.50 (2.12‒2.96)	< 0.001	3.06 (2.58‒3.64)	< 0.001	4.04 (3.36‒4.87)	< 0.001	2.29 (1.78‒2.97)	< 0.001	2.36 (2.00‒2.79)	< 0.001
Unknown	2.94 (2.12‒4.09)	< 0.001	1.78 (1.30‒2.43)	< 0.001	2.23 (1.62‒3.09)	< 0.001	0.98 (0.67‒1.43)	0.92	1.78 (1.30‒2.43)	< 0.001
Occupation										
Farmers	1.00		1.00		1.00		1.00		1.00	
Housewife/unemployed	1.64 (1.50‒1.81)	< 0.001	1.75 (1.59‒1.93)	< 0.001	2.27 (2.05‒2.50)	< 0.001	1.47 (1.29‒1.68)	< 0.001	1.69 (1.54‒1.86)	< 0.001
Others	1.75 (1.57‒1.96)	< 0.001	1.75 (1.57‒1.96)	< 0.001	2.09 (1.86‒2.35)	< 0.001	1.38 (1.18‒1.61)	< 0.001	1.56 (1.40‒1.75)	< 0.001
Parity										
1	1.00		1.00		1.00		1.00		1.00	
2	0.97 (0.88‒1.08)	0.576	1.03 (0.93‒1.14)	0.600	1.08 (0.97‒1.20)	0.141	1.01 (0.88‒1.17)	0.851	1.03 (0.92‒1.14)	0.640
> 3	1.00 (0.89‒1.13)	0.992	1.10 (0.97‒1.24)	0.139	1.17 (1.04‒1.33)	0.010	1.12 (0.95‒1.33)	0.170	1.09 (0.97‒1.23)	0.166

*BCG* Bacillus-Calmette-Guérin, *CI* confidence interval, *cOR* crude odds ratio, *DTP* diphtheria-tetanus-pertussis-containing, *EID* early infant diagnosis, *HEU* HIV-exposed uninfected, *HIV* human immunodeficiency virus, *HepB* hepatitis B, *MCV* measles-containing vaccine, *PHIV* perinatally acquired HIV, children perinatally infected with HIV from mothers

^a^Univariate logistic regression

**Table 7:** Adjusted odds of vaccination factors among HIV-exposed children

Variable	BCG		Polio3		DTP3		HepB3		MCV	
	a*OR* (95% *CI*)	*P*-value^a^	a*OR* (95% *CI*)	*P*-value^a^	a*OR* (95% *CI*)	*P*-value^a^	a*OR* (95%* CI*)	*P*-value^a^	a*OR* (95% *CI*)	*P*-value^a^
EID										
No	1.00		1.00		1.00		1.00		1.00	
Yes	1.86 (1.59‒2.19)	< 0.001	2.50 (2.12‒2.96)	< 0.001	2.86 (2.41‒3.38)	< 0.001	3.17 (2.66‒3.78)	< 0.001	2.47 (2.08‒2.92)	< 0.001
Survival status										
Dead	1.00		1.00		1.00		1.00		1.00	
Live	4.28 (2.91‒6.28)	< 0.001	19.48 (9.08‒41.77)	< 0.001	16.17 (8.46‒30.92)	< 0.001	21.55 (14.41‒32.23)	< 0.001	12.02 (6.30‒22.94)	< 0.001
HIV status										
PHIV	1.00		1.00		1.00		1.00		1.00	
HEU	2.46 (1.88‒3.22)	< 0.001	3.59 (2.68‒4.82)	< 0.001	3.06 (2.31‒4.04)	< 0.001	3.82 (2.91‒5.03)	< 0.001	3.70 (2.74‒5.00)	< 0.001
Unknown	1.63 (1.06‒2.50)	0.026	1.44 (0.87‒2.39)	0.160	1.19 (0.73‒1.93)	0.494	0.66 (0.42‒1.03)	0.066	1.58 (0.95‒2.64)	0.079
Ethnicity										
Minority	1.00		1.00		1.00		1.00		1.00	
Han	2.04 (1.86‒2.23)	< 0.001	1.58 (1.44‒1.73)	< 0.001	1.71 (1.56‒1.88)	< 0.001	1.33 (1.16‒1.52)	< 0.001	1.65 (1.50‒1.81)	< 0.001
Education										
Primary or lower	1.00		1.00		1.00		1.00		1.00	
Middle school	1.73 (1.58‒1.90)	< 0.001	1.87 (1.69‒2.06)	< 0.001	2.20 (2.00‒2.43)	< 0.001	1.62 (1.41‒1.86)	< 0.001	1.68 (1.52‒1.85)	< 0.001
College or above	1.67 (1.40‒1.99)	< 0.001	2.11 (1.74‒2.57)	< 0.001	2.51 (2.03‒3.09)	< 0.001	1.64 (1.24‒2.17)	0.001	1.63 (1.35‒1.96)	< 0.001
Unknown	2.24 (1.59‒3.15)	< 0.001	1.40 (1.00‒1.96)	0.047	1.63 (1.15‒2.31)	0.006	0.84 (0.55‒1.27)	0.402	1.41 (1.01‒1.96)	0.045
Occupation										
Farmers	–		1.00		1.00		–		1.00	
Housewife/unemployed	–	–	1.15 (1.03‒1.28)	0.015	1.39 (1.24‒1.55)	< 0.001	–	–	1.15 (1.03‒1.28)	0.012
Others	–	–	1.11 (0.97‒1.26)	0.121	1.23 (1.07‒1.41)	0.003	–	–	1.05 (0.92‒1.19)	0.456

a*OR* adjusted odds ratio, *BCG* Bacillus-Calmette-Guérin, *CI* confidence interval, *DTP* diphtheria-tetanus-pertussis-containing, *EID* early infant diagnosis, *HEU* HIV-exposed uninfected, *HIV* human immunodeficiency virus, *HepB* hepatitis B, *MCV* measles-containing vaccine, *PHIV* perinatally acquired HIV, children perinatally infected with HIV from mothers; – means not applicable

^a^Multivariate logistic regression using the forward likelihood ratio method

Multivariate analysis showed that HIV-related symptoms were associated with BCG status (a*OR* = 0.86, 95% *CI*: 0.75‒0.99) and MCV status (a*OR* = 0.84, 95% *CI*: 0.72‒0.97). However, co-linearity diagnostics indicated that HIV-related symptoms correlated with survival status (Pearson correlation = 0.287, *P* < 0.05). In the subgroup analysis, HIV-related symptoms showed no difference for BCG or MCV vaccination status in both dead children (BCG: *P* = 0 0.200, MCV: *P* = 1.000) and living children (BCG: *P* = 0.066, MCV: *P* = 0.129). In contrast, survival status had a significant association with BCG or MCV vaccine status, with or without symptoms (*P* < 0.05). Therefore, we excluded HIV-related symptoms from multivariate analysis.

## Discussion

This study is the first to examine immunization coverage in a cohort of HIV-exposed Chinese children. Our study showed low vaccination coverage among children born to HIV-infected women in China. Compared with the WHO-reported Chinese immunization coverage among 1-year-olds (99–100%) for the five examined vaccines [22], HIV-exposed children in this study had a significantly lower coverage level.

The administration of live attenuated vaccines (BCG and MCV) was different in HIV-exposed children from routine practice. For children with PHIV, the WHO recommends that BCG vaccination should be delayed until the infants start antiretroviral therapy (ART) and remain immunologically stable (CD4% > 25%) [23] and that MCV should be routinely administered to potentially susceptible, asymptomatic HIV-infected children [24]. It is also recommended that routine childhood vaccines, whether live-attenuated or killed, should be administered to HEU children. Our study showed that only 52.6% of the HEU group had received the BCG vaccine by their first birthday, which is lower than the figure reported in Latin America and the Caribbean (HEU, 94.2% at 12 months old) [25]. The MCV UTD rate was also lower than previously reported levels (HEU, 94.4% at 4 years old) [26]. Our study also shows that the subjects were under-vaccinated with inactivated vaccines, which should be administered as usual. One explanation for this may be that the HIV-related poor parental practices [27, 28] block the availability of immunization services for children.

We analyzed maternal and child factors associated with insufficient vaccination coverage and found that maternal education remains a significant determinant of immunization, as previous studies have shown that the knowledge gap among uneducated women may be a barrier against immunization [2, 29]. Children of Han ethnicity were more likely to be vaccinated—approximately two times more than those from minority groups—and this may be because the minority groups had poor living conditions and lower socioeconomic status [29, 30]. Our study showed that most children had EID for HIV infection confirmation. Such children had 1.86‒3.17 times greater odds of being vaccinated than those who did not have EID. Limited HIV diagnosis services for HIV-exposed children prevents the implementation of routine immunization [31].

As the diagnosis of HIV in infants is complicated by the passage of maternal HIV antibodies across the placenta, the WHO recommends EID to diagnose HIV infection among children younger than 18 months [32]. In China, HIV-exposed children should undergo EID at 42 days and 3 months of age to confirm HIV status. Meanwhile, Chinese guideline advocate that neonates of unknown HIV status should not be vaccinated with BCG until confirmed to be uninfected, which is stricter than WHO recommendations that state that neonates of unknown HIV status should be vaccinated with BCG if they have no clinical evidence suggestive of HIV infection, regardless of whether or not the mother is receiving ART [24]. We believe that the stricter Chinese recommendation may explain the low coverage of BCG vaccination and the finding that children who had EID were more likely to be vaccinated. Limited laboratory capacity in resource-constrained areas may contribute to why some children did not undergo EID [31].

In our study, HIV-related symptoms had little influence on vaccination status and had interactive effects with survival status. The likely reason for this may be that most of the deceased children died soon after birth without registered HIV-related symptoms and missed immunization. Otherwise, HIV-related symptoms could lead to HIV-related death, and HIV-related symptoms inhibit the uptake of vaccines. Further studies should be performed to investigate the time between the appearance of HIV-related symptoms and child death.

With the increasingly successful use of ART, most HIV-exposed children are not infected; even children with PHIV can have a normal life expectancy. Therefore, attention should be given to low vaccine coverage among children born to HIV-infected women to improve survival in this vulnerable population. Interventions should be developed with a focus on HIV-infected mothers belonging to the uneducated and minority classes. The use of mass media tools may help to improve mothers’ knowledge and understanding of immunization. PMTCT services should be integrated with immunization services in the future. Particularly, more attention should be paid to EID, and the government needs to optimize recommendations for the immunization of HIV-exposed children.

Our study had some limitations. First, the data were collected from the Chinese information system for PMTCT. This may be biased by low incidence reporting due to under-detection, misclassification, and underreporting because data collection on HIV-exposed children relies on health facility reporting. Second, there have been dramatic changes from the “basic” antigens that were used to define a fully vaccinated child in the early 1980s [20]: the new combined acellular pertussis, diphtheria, tetanus, inactivated poliomyelitis, and *Haemophilus influenzae* type b conjugate vaccine has become popular recently, which may lead to misunderstanding and misreporting of the vaccination status. Third, we did not consider the factors associated with healthcare providers. Healthcare workers who are responsible for child immunization may miss opportunities for immunizing HIV-infected children. This may be because they are unaware of vaccination recommendations in this population and are therefore overly concerned about possible risks associated with the use of vaccines [26, 29, 30] or because of limited EID services for confirming children’s HIV status.

## Conclusions

Chinese HIV-exposed children had low vaccination coverage. PHIV children were significantly less likely to be vaccinated than HEU children. Interventions to address this should be developed with a focus on minority children whose mothers do not have formal education. Particularly, more attention should be paid to EID to increase access to immunization. With the risk of mother-to-child transmission of HIV reduced to 5% or less, children can achieve the expected lifespan with effective treatment. More attention should be paid to the neglected immunization problem. We also strongly encourage further research on strategies to improve routine vaccination for HIV-exposed children.

## Data Availability

The dataset supporting the conclusion of this article is available upon reasonable request from the corresponding author.

## Abbreviations

a*OR*: Adjusted odds ratio
ART: Antiretroviral therapy
BCG: Bacillus Calmette-Guérin
*CI*: Confidence interval
c*OR*: Crude odds ratio
DTP: Diphtheria-tetanus-pertussis-containing
EID: Early infant diagnosis
HEU: HIV-exposed uninfected
HIV: Human immunodeficiency virus
HepB: Hepatitis B
MCV: Measles-containing vaccine
PHIV: Perinatally acquired HIV
PMTCT: Prevention of mother-to-child transmission
UTD: Up to date
WHO: World Health Organization
